# Comparative absorption of curcumin formulations

**DOI:** 10.1186/1475-2891-13-11

**Published:** 2014-01-24

**Authors:** Ralf Jäger, Ryan P Lowery, Allison V Calvanese, Jordan M Joy, Martin Purpura, Jacob M Wilson

**Affiliations:** 1 Increnovo LLC, 2138 E Lafayette Pl, Milwaukee, WI 53202, USA; 2 Department of Health Sciences and Human Performance, The University of Tampa, Tampa, FL 33606, USA

**Keywords:** Curcumin, Absorption, Bioavailability

## Abstract

**Background:**

The potential health benefits of curcumin are limited by its poor solubility, low absorption from the gut, rapid metabolism and rapid systemic elimination. The purpose of this study was the comparative measurement of the increases in levels of curcuminoids (curcumin, demethoxycurcumin, bisdemethoxycurcumin) and the metabolite tetrahydrocurcumin after oral administration of three different curcumin formulations in comparison to unformulated standard.

**Methods:**

The relative absorption of a curcumin phytosome formulation (CP), a formulation with volatile oils of turmeric rhizome (CTR) and a formulation of curcumin with a combination of hydrophilic carrier, cellulosic derivatives and natural antioxidants (CHC) in comparison to a standardized curcumin mixture (CS) was investigated in a randomized, double-blind, crossover human study in healthy volunteers. Samples were analyzed by HPLC-MS/MS.

**Results:**

Total curcuminoids appearance in the blood was 1.3-fold higher for CTR and 7.9-fold higher for CP in comparison to unformulated CS. CHC showed a 45.9-fold higher absorption over CS and significantly improved absorption over CP (5.8-fold) and CTR (34.9-fold, all p < 0.001).

**Conclusion:**

A formulation of curcumin with a combination of hydrophilic carrier, cellulosic derivatives and natural antioxidants significantly increases curcuminoid appearance in the blood in comparison to unformulated standard curcumin CS, CTR and CP.

## Background

Curcumin (diferuloylmethane; 1,7-bis[4-hydroxy-3-methoxyphenyl]-1,6-heptadiene-3,5-dione) is the major bioactive component of the spice herb turmeric or *Curcuma longa* L., a widely used natural food product in curry powder and food coloring (mustard). While used traditionally in Indian and Chinese medicine and widely consumed in the Asian diet, recent clinical studies have demonstrated its benefits in several health ailments including cancer [1,2], immune deficiencies [3], cardiovascular health [4], Alzheimer’s [5], diabetes [6], arthritis [7] and Crohn’s disease [8], despite having a low bioavailability [9]. Curcumin has been shown to increase vasodilation similar to exercise [10] and to curcumin ingestion with aerobic exercise training is more effective than curcumin ingestion or aerobic exercise training alone [11]. Curcumin works by way of modulating multiple molecular targets, cell signaling proteins, cell cycle proteins, cytokines and chemokines, enzymes, receptors and cell surface adhesion molecules [12,13]. As a polyphenolic antioxidant, curcumin has been shown to have neuroprotective [14] and anti-inflammatory properties [15]. Commercially available natural curcumin is a mixture of three curcuminoids: curcumin (ca. 75%), demethoxycurcumin (ca. 15%), and bisdemethoxycurcumin (ca. 5%) [16].

**Figure 1:**
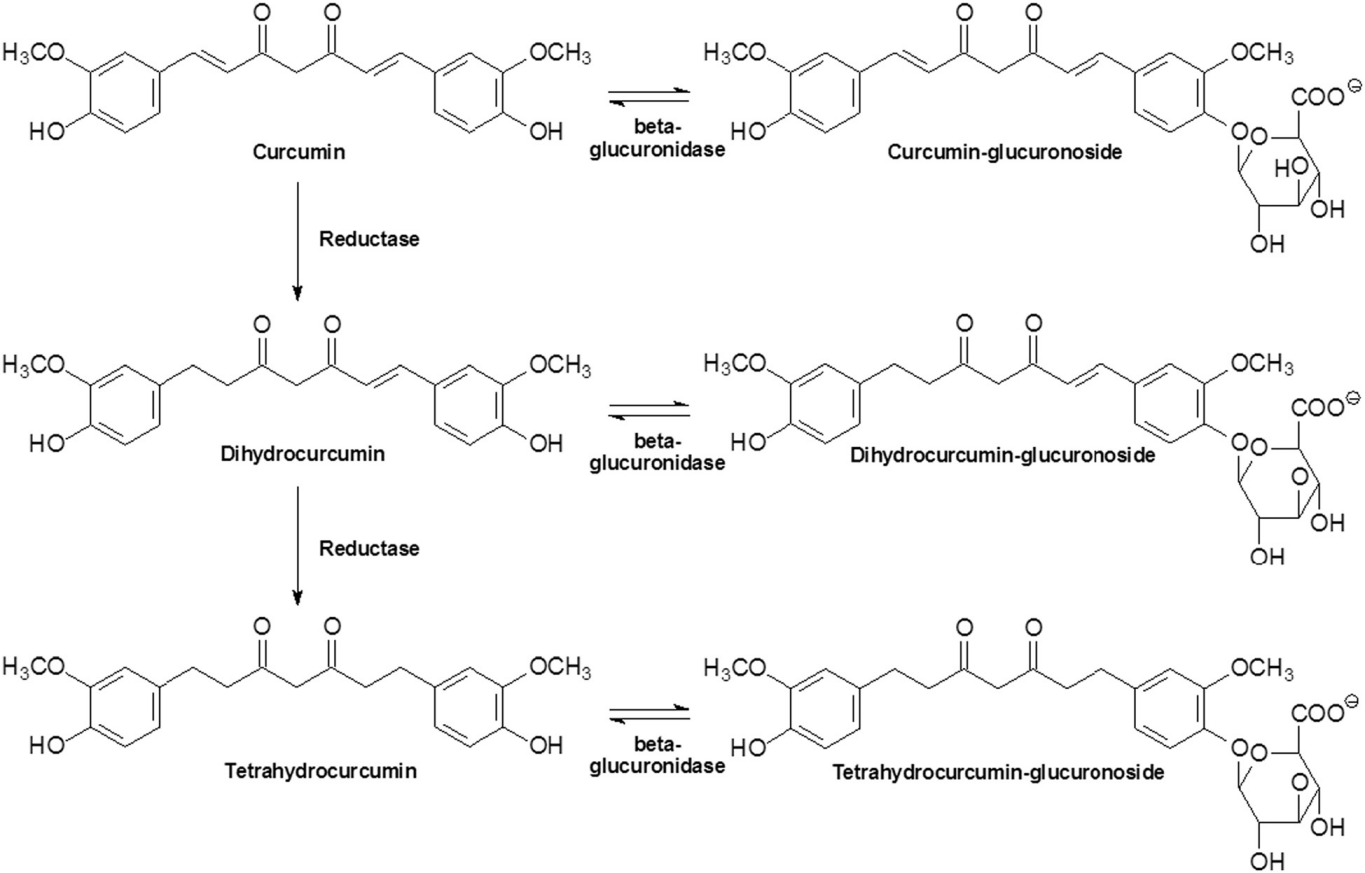
**Metabolic pathway of orally ingested curcumin **[19]**.**

However despite its demonstrated effects, the potential health benefits of curcumin are limited by its poor solubility, low absorption from the gut, rapid metabolism and rapid systemic elimination [17]. While the major portion of ingested curcumin is excreted through the feces unmetabolized, the small portion that is absorbed is extensively converted to its water-soluble metabolites, glucuronides and sulfates. Microbial metabolism of curcumin yields dihydrocurcumin, and tetrahydrocurcumin through NADPH dependent reduction. Curcumin and its reduced metabolites are conjugated with monoglucuronide via beta-glucuronidase, a monosulfate via sulfatase enzymes and a mixed sulfate/glucuronide, resulting in curcumin-glucuronoside, dihydocurcumin glucuronoside, tetrahydrocurcumin glucuronoside or corresponding monosulfate and mixed sulfate/glucuronoside (see Figure 1) [18,19].

Different strategies have been pursued to improve the absorption of curcumin including nanocrystals, emulsions, liposomes, self-assemblies and nanogels [20]. In animals, co-administration of curcumin with an extract obtained from the black pepper has been shown to increase the absorption (AUC) of curcumin by 1.5-fold. Whereas, a complex of curcumin with phospholipids increased absorption by 3.4-fold [21] and a formulation of curcumin with a micellar surfactant (polysorbate) has been shown to increase the absorption of curcumin in mice 9.0-fold [22]. A micro emulsion system of curcumin, which consists of Capryol 90 (oil), Cremophor RH40 (surfactant), and Transcutol P aqueous solution (co-surfactant) has been shown to increase the relative absorption in rats by 22.6-fold [23]. Polylactic-co-glycolic acid (PLGA) and PLGA-polyethylene glycol (PEG) (PLGA-PEG) blend nanoparticles increased curcumin absorption by 15.6- and 55.4-fold, respectively, compared to an aqueous suspension of curcumin in rats [24].

Food-grade formulations to enhance the absorption of curcumin have been studied in human clinical trials [25,26]. A proprietary formulation of curcumin has been developed retaining and utilizing more components of the raw turmeric root which are usually eliminated during extraction. The combination of curcuminoids and volatile oils of turmeric rhizome (CTR) resulted in a 6.9-fold increase in human absorption of curcumin [25]. The inclusion of curcumin in a lipophilic matrix (Phytosomes, Curcumin:Soy Lecithin:Microcrystalline Cellulose 1:2:2, CP) has been shown to increase the relative human absorption of curcumin by 19.2-fold [26]. A formulation made by mixing curcumin with glycerin, gum ghatti, and water, followed by wet milling and dispersion by high-pressure homogenization has been shown the increase curcumin appearance in the blood by 27.6-fold [27]. A novel curcumin formulation which was made water soluble by dispersing curcumin and antioxidants (tocopherol and ascorbyl palmitate) on a water-soluble carrier such as polyvinyl pyrrolidone has been shown to have greater antidepressant action compared to conventional curcumin [28].

**Figure 2:**
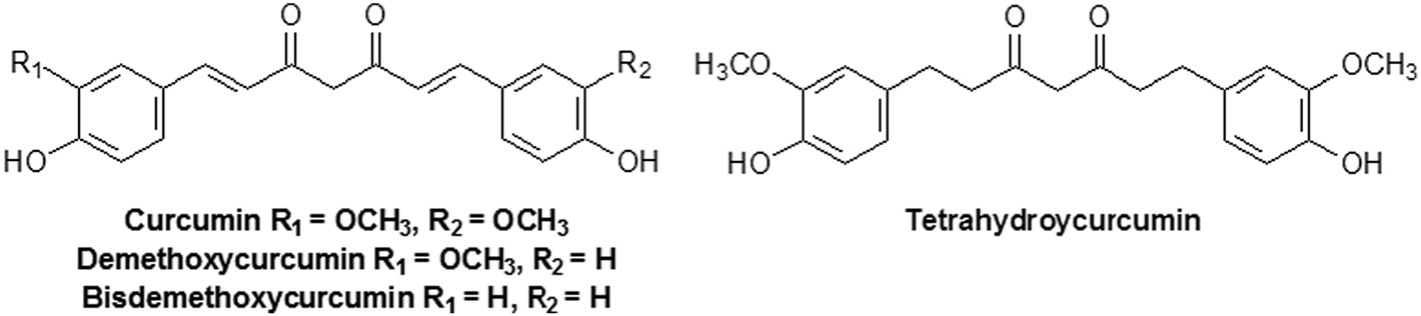
Chemical structure of curcuminoids and the metabolite tetrahydrocurcumin.

The purpose of this study was the comparative measurement of the increases in levels of curcuminoids (curcumin, demethoxycurcumin, bisdemethoxycurcumin) and the metabolite tetrahydrocurcumin (see Figure 2) in plasma after the administration of a single dose of the above mentioned novel water soluble curcumin formulation containing turmeric extract 20-28%, a hydrophilic carrier 63-75%, cellulosic derivatives 10-40% and natural antioxidants 1-3% (CHC), in comparison to CP, CTR and standard curcumin (CS) in healthy volunteers.

## Methods

### Subjects

Fifteen subjects were recruited for this study of which twelve subjects (11 male, 1 female; age 23.0 ± 2.4 years; height 182.9 ± 6.1 cm; weight 86.2 ± 4.2 kg; 1 African American and 11 Caucasian) completed the study. One subject never started the study and the other two drop-outs occurred due to personal reasons. One drop-out was caused because the subject was feeling faint during the blood draw and was instructed to not continue to avoid a syncope episode, and the second drop out was due to a lack of compliance with the protocol. The protocol was approved by The University of Tampa Institutional Review Board and the study was registered with Current Controlled Trials (ISRCTN28011391).

### Study materials

CP (lot number 30857/M3) was acquired from Indena USA Inc., Seattle, WA, USA. CTR (lot number 1206/B-16) was acquired from DolCas Biotech, LLC, Landing, NJ, USA. CS (lot number C90680) was acquired from Sabinsa Corporation, East Windsor, NJ, USA. And CHC (lot number CU20DNS1-008/009) was provided by OmniActive Health Technologies, Inc., Morristown, NJ, USA. An inert filler (microcrystalline cellulose) was used to match the total weight of each of the study materials. Subjects consumed optically identical 6 hard gel capsules of each of the study materials per setting yielding 376 mg of total curcuminoids for CHC, CTR and CP and 1,800 mg of total curcuminoids for CS. The dose was selected based on Cuomo et al. [26].

### Study procedure

Prior to testing, each volunteer underwent screening and the consent visit to ensure eligibility and voluntary willingness to participate. Following consent, each volunteer completed 4 trials with 9 blood draws each in a randomized, double‒blinded order separated by 7 days. During each trial, each volunteer reported to the laboratory in the morning between 6:00 and 10:00 hours following a 10‒hour overnight fast (except for water). Subjects enrolled in the study needed to meet the following inclusion parameters: 20-35 years of age; have not been consuming any curcumin-containing supplements or foods for two weeks prior to testing; no history of any of the following: gastrointestinal problems, gallbladder issues, hyperacidity, gastric/duodenal ulcers; no use of NSAIDS or any blood thinners/anti-thromobic agents; no prior use of H2 blockers, proton pump inhibitors or blood sugar-lowering agents; non-diabetic, non-hyperglycemic, non-hemophiliac; and no known allergies to soy.

**Figure 3:**
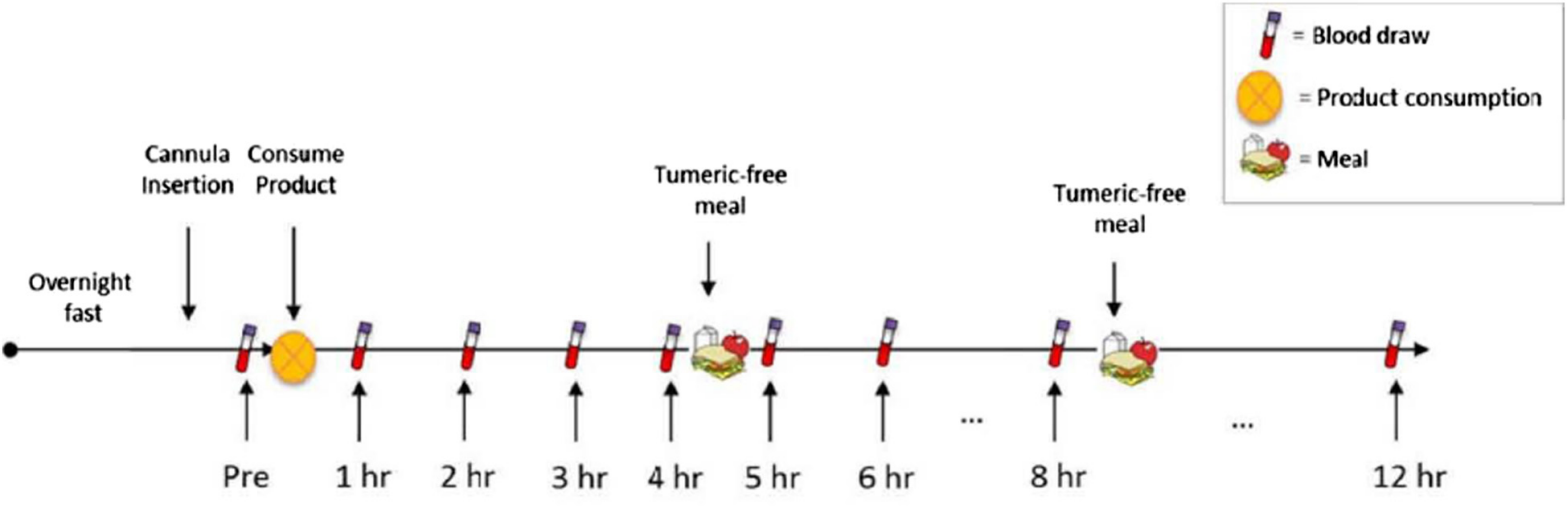
Schematic representation of the study procedure.

During each visit, the volunteer was seated comfortably while a catheter was introduced into a forearm vein by a qualified phlebotomist. After equilibration, a baseline blood sample was collected and one of four treatment dosages of curcumin was consumed with water. Blood samples were then drawn at 1, 2, 3, 4, 5, 6, 8 and 12 hours intervals following product consumption (see Figure 3). After the 4‒hour and 8‒hour blood samples had been drawn, a turmeric‒free standardized meal was provided. The first meal consisted of 40 g chocolate whey protein isolate and 80 g instant oatmeal dissolved in 30 mL of water plus 473 mL of water to drink. The second meal consisted of 230 g turkey breast, 2 slices of whole wheat bread, 15 g light miracle whip, 170 g of fat free Greek yogurt and 473 mL of water to drink. Each subsequent trial was separated by at least 7 days as a washout period and followed identical study procedures, except for the consumption of a different curcumin formulation. Product formulations were blinded to both the investigators and the volunteers and coded so that neither knew which formulation is consumed during each trial.

### Sample collection

During each timepoint, 6 mL of blood were drawn off the catheter into vacutainer tubes. Blood tubes were centrifuged at 2000×g for 10 minutes and plasma was aliquoted into eppendorf storage until analysis. Plasma samples were stored in a ‒80°C freezer until analysis and thawed only once prior to avoid degradation.

### Sample preparation

The sample preparation was performed in accordance with Cuomo et al. [26]. A 0.2 mL aliquot of plasma was transferred to a clean microcentrifuge tube and next treated with 100 μL of a solution containing 1000 U of β-Glucuronidase/sulfatase (EC 3.2.1.31) from *Helix pomatia* (Sigma, St. Louis, MO) in 0.1 M phosphate buffer (pH 6.86). The resulting mixture was then thoroughly vortexed and incubated at 37°C for 1 hour to hydrolyze the phase-2 conjugates of curcuminoids. After incubation, curcuminoids were extracted with 1 mL of ethyl acetate, and the mixture was vortexed for 1 minute, followed by sonication in a water bath for 15 minutes. After centrifugation at 15,000 g for 6 minutes, the upper organic layer was transferred to a 2 mL microcentrifuge tube and evaporated to dryness at 30°C under negative pressure in a centrifugal concentrator. This process was repeated for a total of two extractions. This solution concentration was 50 ng/ul. The dried extract was reconstituted in 100 μL of methanol, and 10 μL was injected into the HPLC-MS/MS. An internal standard “Salbutamol” (ISTD) was prepared and used to ensure data accuracy. The standard curcuminoids for quantitation were obtained from Sigma Aldrich, USA.

### Chromatographic analysis of the curcuminoids

The blood plasma samples were evaluated for curcumin, demethoxycurcumin, and bisdemethoxycurcumin [26] and tetrahydrocurcumin [21] by tandem mass spectrometry detection (HPLC/MS/MS). Prior to the actual study a case study was performed to validate and the analytical method. A six-point calibration curve was created by plotting the peak area ratio (y) of curcumin to internal standard versus the curcumin concentration. The regression parameters were calculated using the MassHunter Workstation Software (Agilent Technologies, Santa Clara, CA). The calibration curves were linear in human plasma with curves of y = 1.24x (r = .99) and y = 0.58x (r = 0.99) for curcumin and tetrahydrocurcumin, respectively. The accuracy of curcumin and tetrahydrocurcumin in the control sample was 92-100% and 101-105%, respectively, with a coefficient of variation of 5.7 and 3.7%, respectively. The analytical method was able to detect curcumin, demethoxycurcumin, bisdemethoxycurcumin and tetrahydrocurcumin in human plasma and is very accurate and reliable. The Internal Standard “Salbutamol” (ISTD) was prepared by adding 5.0 mg to 100 ml of Methanol in a volumetric flask then vortex. This solution concentration was 50 ng/ul. HPLC-MS-MS: Agilent 1290 HPLC system with an Aglient 6460 tandem mass spectrometer with ESI source. Column: Kinetex XB-C18 100 Å, 2.1×50 mm, 2.6 micron. Pre-column: security guard ultra, C18, 2.1 mm. Temperature in column chamber was set to 50°C. The mass spectrometer was run in the multiple reaction monitoring (MRM) mode and the transitions monitored were *m/z* 373.2 → 137.1 for tetrahydrocurcumin, 369.1 → 285.1 for curcumin, 339.1 → 255.1 for demethoxycurcumin, and 309.1 → 225.0 for bisdemethoxycurcumin.

### Statistical analysis

The population pharmacokinetics following the oral administration of the curcumin formulations were assessed by a Non-linear Mixed Effects Model using SPSS 21.0 (SPSS Inc., Chicago, IL). All plasma concentrations were log-transformed by use of natural logarithms and analyzed for meeting assumptions before proceeding with analysis. This two-stage model approach evaluates the fixed effects that demonstrate the bioavailability parameters of the four curcuminoids across the population for whom the curcumin formulations are intended and the random effects denote the variability of plasma concentrations across the subjects from the entire population. The fixed effects that demonstrate the bioavailability parameters in the population were included as the interaction in metabolic processes of the four curcuminoids over the time sampling hours 1-12 hours, specific to each curcumin formulation. The random effects were included to account for the auto-correlation of residuals in the extent of bioavailability across the different curcumin formulations in the same subjects. Plasma concentrations of all curcuminoids k (k = 1, . . . 4) measured for the individual subject *i* (*i* = 1, . . . N) at each time sampling hour *j* (*j* = 1, . . . 8) was further characterized into a vector C*kij* with the curcumin formulations compared in separate levels over the duration of the study. Mean plasma concentration time curves were obtained by taking the antilogarithm of the mean predicted plasma concentration during each time point for the individual curcuminoids across the formulations. The *c*_max_ was the maximum observed plasma concentration directly from the mean plasma concentration time profile and the Area Under the Plasma Concentration Time-Curve (AUC) was calculated by the definite integral from 0-12 hours of the mean plasma concentration time-curves. Calculation of *t*½ could not take place as a number of the formulations did not decline in concentration over the 12 hour time period.

## Results

Absorption of curcumin, demethoxycurcumin, bisdemethoxycurcumin, and appearance in the blood of tetrahydrocurcumin was measured in 12 healthy volunteers (n = 12) in a randomized, double-blind, crossover study. The subjects consumed either 376 mg of total curcuminoids in the form of CP, CTR, or CHC, or 1,800 mg of the corresponding non-formulated CS in accordance with Cuomo et al. [26] Since free curcumin could not be detected in previous studies [26], even at levels of up to 12,000 mg [29], plasma samples were treated with *Helix pomatia* glucuronidase/sulfatase before HPLC-MS/MS analysis (for experimental details, see the Experimental Section). All four treatments were well tolerated and no adverse events were reported.

**Figure 4:**
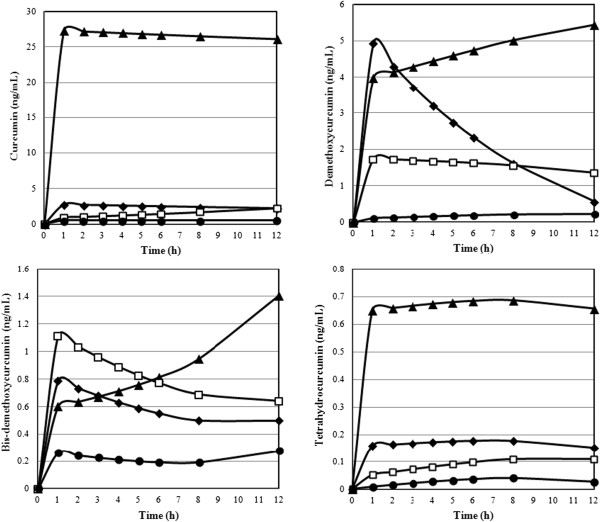
**Plasma concentrations time-curves for curcumin, demethoxycurcumin, bisdemethoxycurcumin, and tetrahydrocurcumin for ▲ CHC** ♦ **CP****CS ● CTR formulations.** Concentrations are expressed in ng/mL and refer to enzymatically hydrolyzed plasma samples.

**Figure 5:**
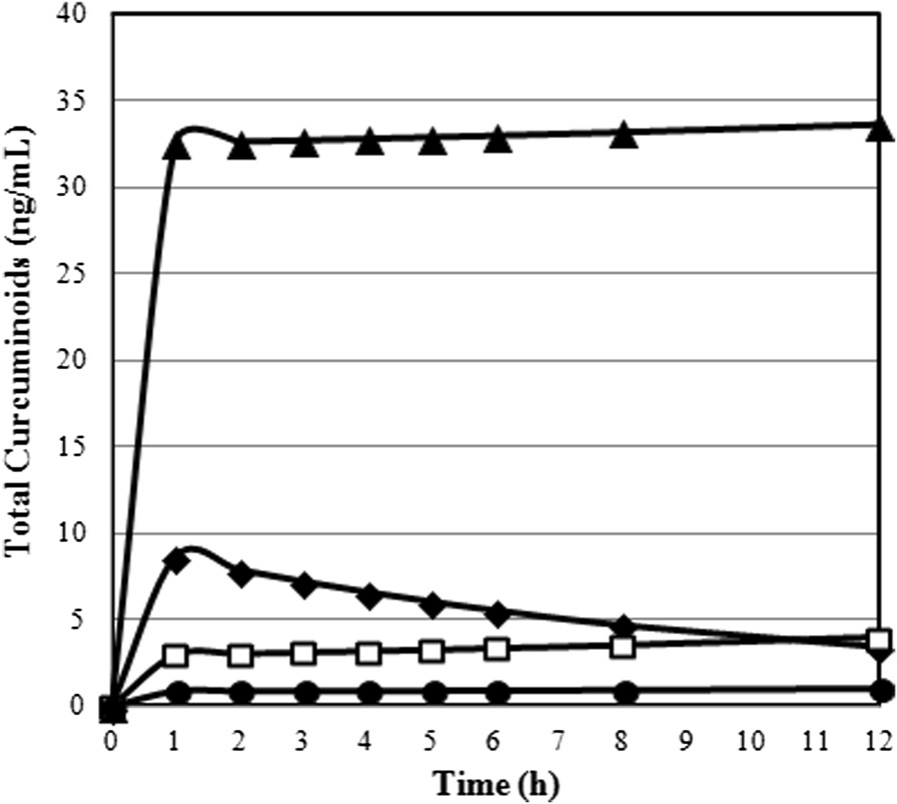
**Plasma concentration time-curve for total curcuminoids in the validated HPLC-MS/MS assay of plasma samples for ▲ CHC** ♦ **CP****CS ● CTR formulations after oral administration of 376 mg of total curcuminoids for CHC, CTR and CP and 1,800 mg of total curcuminoids for CS.** Concentrations are expressed in ng/mL and refer to the total amount of all four curcuminoids.

Pharmacokinetic data of the individual curcuminoids for the formulation were each plotted on a plasma concentration vs. time-curve (Figures 4 and 5). Area Under the Plasma Concentration Time-Curve (AUC), c_max_, t_max_ and relative absorption (F) were calculated for each curcuminoid at all levels of the formulations and are presented in Table 1. The relative absorption was calculated by dividing the value of test product (CTR, CP or CHC) by the value of reference product (CS) multiplied by the dosage of the reference product (1,800 mg) divided by dosage of the test product (376 mg).

**Table 1:** **Pharmacokinetic parameters of curcuminoid concentrations Area Under the Curve (AUC), *c***_**max**_**, *t***_**max**_**, and relative absorption for each formulation**

**Curcuminoid**	**Formulation**	**AUC**_**0-12 **_**(ng/mL•hr)**	**C**_**max **_**(ng/mL)**	**t**_**max **_**(h)**	**Relative Absorption (F)**
Curcumin	CS	10.8 ± 1.7^x^	2.3 ± 0.3	7.4 ± 1.0^^^	1.0
	CTR	5.8 ± 0.1	0.5 ± 0.0	3.2 ± 1.0	2.6
	CP	28.7 ± 2.6^+^	2.8 ± 0.3	1.7 ± 1.0	12.7^+^
	CHC	307.6 ± 44.6^#^	27.3 ± 6.4^#^	1.4 ± 0.5	136.3^#^
Demethoxycurcumin	CS	18.4 ± 2.3	1.7 ± 0.2^x^	1.2 ± 0.1	1.0
	CTR	2.2 ± 0.5	0.2 ± 0.1	8.1 ± 0.2^^&^	0.6
	CP	28.0 ± 3.3^x^	5.0 ± 0.7^+^	1.0 ± 0.1	7.3^+^
	CHC	54.5 ± 8.1^+^	5.4 ± 1.1^+^	11.9 ± 0.1^^&^	14.2^#^
Bisdemethoxycurcumin	CS	9.3 ± 0.8^x^	1.1 ± 0.1^x^	1.3 ± 1.1	1.0
	CTR	2.6 ± 0.4	0.3 ± 0.1	11.9 ± 0.7^^&^	1.3
	CP	6.7 ± 0.3^x^	0.8 ± 0.1^x^	1.3 ± 0.2	3.5^+^
	CHC	10.2 ± 1.3^x^	1.4 ± 0.2^x^	11.9 ± 1.2^^&^	5.3^#^
Tetrahydrocurcumin	CS	1.1 ± 0.1	0.1 ± 0.0	8.1 ± 1.1	1.0
	CTR	0.3 ± 0.1	0.0 ± 0.0	8.2 ± 1.0	1.3
	CP	1.9 ± 0.2	0.2 ± 0.0^x^	8.0 ± 1.2	8.3^+^
	CHC	7.7 ± 0.6^#^	0.7 ± 0.0^#^	8.0 ± 0.8	33.5^#^
Total Curcuminoids	CS	39.6 ± 1.5^x^	5.2 ± 0.2^x^	9.5 ± 0.2^x^^	1.0
	CTR	10.9 ± 0.4	1.1 ± 0.1	1.8 ± 0.7	1.3
	CP	65.3 ± 2.3^+^	8.7 ± 0.4^+^	1.7 ± 0.4	7.9^+^
	CHC	380.0 ± 23.9^#^	34.9 ± 3.3^#^	1.7 ± 0.4	45.9^#^

Data expressed as geometric (AUC, c_max_) or arithmetic (t_max_) mean ± standard errors. ^#^significantly different (p < 0.05) compared to CS, CTR, and CP. ^+^significant different (p < 0.05) compared to CS and CTR. ^x^significantly different (p < 0.05) compared to CTR. ^^^significantly different (p < 0.05) compared to CP. ^&^significantly different (p < 0.05) compared to CS.

There were significant differences between the time of maximum plasma concentrations (t_max_) of the four products as shown by the results of a nonparametric Friedman’s Test (χ^2^ = 8.5 p < 0.05). Post-hoc tests of a Wilcoxon Signed Rank Test displayed that CTR had a significantly higher t_max_ in comparison to CP (Z = -2.53 p < 0.05). Relative total curcuminoid appearance was 7.9-fold higher for CP in comparison to the unformulated CS product. CHC showed a 45.9-fold higher relative appearance over standard and was significantly improved over CS, CP and CTR.

## Discussion

The purpose of this study was to investigate the effects of a novel formulation of curcumin (CHC) in comparison to unformulated standard curcumin (CS) and two formulations previously shown to improve the absorption of curcuminoids (CP and CTR). The novel finding in the present study is that CHC significantly increased curcuminoid appearance in the blood in comparison to CS, CP and CTR. The 45.9-fold increased oral absorption of the CHC formulation as compared with the CS formulation is based on an increased solubility of the CHC formulation. The solubility was enhanced by dispersing a highly purified powder [with min 95% curcuminoids] in a water-soluble carrier (polyvinyl pyrrolidone) along with other encapsulating agents. Tocopherol and ascorbyl palmitate were used to prevent degradation of curcumin.

Certain health benefits associated with curcuminoids may depend on the amount and presence of methoxy groups and their effect on the phenyl ring indicating that curcumin might be the most potent individual curcuminoid. The antioxidant potency of curcuminoids decreases with a decrease in the number of methoxy groups (curcumin > demethoxycurcumin > bisdemethoxycurcumin) [30]. In addition, the antiulcer potency [31] and anti-inflammatory activity [32] of curcumin is stronger than that of demethoxycurcumin, whereas the methoxy groups play a minor role in the growth-modulating effects of curcuminoids [32]. Cuomo et al. [26] reported that the phospholipids in CP increase the appearance in the blood of demethoxylated forms of curcumin. Standard curcumin contains 4 times the amount of curcumin in comparison to demethoxycurcumin; however, the formulation with phospholipids results in demethoxycurcumin being the major plasma curcuminoid for CP, and not curcumin. The current study showed increased appearance in the blood of demethoxycurcumin for CP in comparison to curcumin and their natural ratio in the test product, whereas curcumin is the major plasma curcuminoid for CTR and CHC, which based on the desired health benefits of curcumin administration, would be the preferred profile.

Tetrahydrocurcumin plays an important role in the antioxidant mechanism of curcumin and has been shown to be the most potent antioxidant of the curcuminoids measured in this study [33]. In addition, tetrahydrocurcumin has been reported to have health promoting benefits. It has been shown to have greater anti-inflammatory potency than curcumin in carrageenan-induced paw edema [34]. Plasma concentrations of the metabolite tetrahydrocurcumin were lower than the concentrations of the three curcuminoids present in the study materials administered. CHC showed the highest concentrations of tetrahydrocurcumin, followed by CP, CS and CTR, matching the order of plasma curcumin concentrations.

This study and Cuomo et al. [26] showed several differences in study design. Cuomo et al. measured plasma concentrations of the samples over a 24 hour period of time compared to 12 hours as demonstrated in this study and subjects in this study were fasted while Cuomo et al. gave a high fat meal with the curcumin administration which has shown to slow the mean transit time (MTT) in the gastrointestinal tract and also improve the absorption of fat soluble ingredients. Additional differences include the fact that Cuomo et al. did not analyze the concentration of tetrahydrocurcumin in the blood plasma and did not use an internal standard. In this study an internal standard, “Salbutamol” (ISTD), was used to improve accuracy and reliability of the data outcome as previously described by Liu et al. 2006 in an absorption study in rats [21]. Due to the differences in design absolute values cannot be directly compared.

Antony et al. studied the effects of curcumin-lecithin-piperine (CTR) or a curcumin control in 11 healthy subjects in a cross-over design with a two week wash-out period [25]. The analytical measurement did not use an internal standard and only determined the curcumin content in the blood for up to 8 hours after administration. The study showed a 6.9-fold increased absorption over control. Our study showed an approximately 30% increased relative absorption of CTR. In 2006, Lao et al. studied the safety and appearance in the blood of a single dose of CS, the same material we used as control in our study [29]. Twenty four healthy volunteers (n = 24) consumed escalating single doses of 500, 1,000, 2,000, 4,000, 6,000, 8,000, 10,000 and 12,000 mg of CS. No curcumin was detected in serum at up to 8 g of CS. Two subjects (one taking 10,000 mg, and the other taking 12,000 mg) showed low levels of curcumin whereas no plasma concentrations of curcumin were detected in the remaining subjects at the 10,000 or 12,000 mg dose levels.

The absolute values of other studies cannot be compared with the results of our study due to differences in subjects, analytical method, study design and administration of the product. The present study is the first and only study which measured the constituent parts of the curcumin formulation derived from the extraction process (curcumin, bisdemethoxycurcumin and demethoxycurcumin) and the major metabolite of orally ingested curcumin (tetrahydrocurcumin).

One limitation in the study design was the sampling time frame. Our data indicated that the curcumin half-life was estimated to be 6-7 hours and that the plasma levels of the conjugated curcuminoids were not in their elimination phase. Thus, while we sampled from 0-12 hours, we propose future research to assess a 24 hour sampling period.

## Conclusion

A formulation of curcumin with a combination of hydrophilic carrier, cellulosic derivatives and natural antioxidants significantly increases curcuminoid appearance in the blood in comparison to unformulated standard curcumin CS (45.9-fold), CTR (34.9-fold) and CP (5.8-fold).

## Competing interests

The authors declare that they have no competing interests.

## Authors’ contributions

The manuscript was written through contributions of all authors. All authors have given approval to the final version of the manuscript.
